# Mutation analysis of common deafness genes among 1,201 patients with non‐syndromic hearing loss in Shanxi Province

**DOI:** 10.1002/mgg3.537

**Published:** 2019-01-28

**Authors:** Yongan Zhou, Chao Li, Min Li, Zhonghua Zhao, Shuxiong Tian, Hou Xia, Peixian Liu, Yaxin Han, Ruirui Ren, Jianping Chen, Caihong Jia, Wei Guo

**Affiliations:** ^1^ Shanxi Medical University Second Affiliated Hospital Taiyuan Shanxi China; ^2^ The Graduate School Shanxi Medical University Taiyuan Shanxi China; ^3^ Institute of Biomedical Sciences Shanxi University Taiyuan Shanxi China; ^4^ Xinzhou Traditional Chinese Medicine Hospital Xinzhou Shanxi China

**Keywords:** gene, *GJB2*, *mtDNA 12S rRNA*, non‐syndromic hearing loss, *SLC26A4*

## Abstract

**Background:**

Hearing impairment is one of most frequent birth defects, which affects nearly 1 in every 1,000 live births. However, the molecular etiology of non‐syndromic deafness in China is not well studied. Here, we have investigated the presence of mutations in three genes commonly mutated in non‐syndromic deafness patients in Shanxi Province, which has the highest frequency of birth defects in China.

**Methods:**

In total, 1,201 unrelated non‐syndromic deafness patients and 300 healthy individuals were enrolled. The hearing ability was confirmed by audiologic evaluation. Three major deafness‐related genes (*GJB2, SLC26A4 (PDS), *and *mtDNA 12S rRNA*) of all individuals enrolled were analyzed by Sanger sequencing.

**Results:**

The results showed that *GJB2* mutations accounted for 21.23% (255/1,201) in the patient group, with c.235delC, a hotspot mutation, accounting for 10.99% (132/1,201). Moreover, 11 new *GJB2* mutations were identified. *SLC26A4* mutations accounted for 9.33% (112/1,201) in the patient group, with IVS7‐2A>G as the most prevalent mutation accounting for 4.75% (57/1,201). In addition, 15 patients (1.25%) were found to carry *mtDNA 12S rRNA* c.1555A>G mutation, while only two cases had the *mtDNA 12S rRNA* c.1494C>T.

**Conclusion:**

In our research, it was found that c.235delC in *GJB2* and c.919‐2A>G (IVS7‐2A>G) in *SLC26A4* were the highest frequency pathogenic variants in Shanxi Province. Taken together, our data will enrich the database of deafness mutations and will help clinical diagnosis, treatment, and genetic counseling of hearing impairment.

## 1 INTRODUCTION

Hearing impairment is one of the most common sensory disorders; it affects about 70 million people around the world, and notably, almost one live birth in every 1,000 newborns has hearing impairment. More than half of childhood hearing impairment is caused by genetic defects. Among these, 30% of cases are syndromic with other abnormalities, while approximately 70% are non‐syndromic, in which the hearing impairment is the only distinctive clinical feature in these patients (Nance, Lim, & Dodson, 2006; Qing et al., 2015).

Since 1993, when the first hearing loss gene was identified, more than 100 loci for deafness genes have been mapped and about 80 genes have been reported to be associated with non‐syndromic hearing loss (*Hereditary Hearing Loss Homepage*). Exhilaratingly, combining the GWAS analysis and the next‐generation sequencing technique, more than 150 loci have been discovered to date, including 54 autosomal dominant loci, 71 autosomal recessive loci, 5 X‐linked loci, two modifiers, and 1 Y‐linked locus (Wu et al., 2017). Although many new genes have been found, the most common genes associated with hearing impairment still are *GJB2, SLC26A4,* and *mtDNA 12S rRNA*.

Mutations in gap junction beta‐2 gene (*GJB2,* MIM #121011) account for 50% of autosomal recessive hearing loss (HL) in most populations in the world (Palmada et al., 2006; Wiley, Choo, Meinzen‐Derr, Hilbert, & Greinwald, 2006). The *GJB2 *gene, expressed in the lateral wall and the supporting cells of the organ of Corti in the inner ear, encodes connexin 26, a gap junction protein that facilitates potassium ion transport (Locke et al., 2007; Zhao & Yu, 2006). More than 110 different mutations have been identified in *GJB2*, of which the c.35delG mutation shows the highest frequency in the Caucasian population while c.235delC is highest in Southeast Asians (Yan et al., 2017).

The mutations in *SLC26A4 *(MIM #605646) are the second most frequent cause of non‐syndromic hearing loss. Mutations in *SLC26A4* are normally associated with large vestibular aqueduct syndrome, showing congenital temporal bone malformation of the vestibular aqueduct as well as Mondini dysplasia (Chattaraj et al., 2017; He et al., 2017; Ma et al., 2016). Avoidance of head and ear trauma in individuals with *SLC26A4 *mutations would dramatically reduce the incidence of deafness. c.919‐2A>G is the most frequent mutation in *SLC26A4* gene.

Mutations of mitochondrial DNA (*mtDNA*) are associated with maternally inherited sensorineural hearing loss. The *mtDNA* of human is double‐stranded, encoding 13 protein subunits, two ribosomal RNAs (rRNAs), and 22 transfer RNAs (tRNAs) (Alves, Calmeiro, Calmeiro, Macario, & Silva, 2017; Mutai, Watabe, Watabe, Kosaki, Ogawa, & Matsunaga, 2017). The c.1555A>G is the most frequent mutation in *mtDNA* associated with HL.

Early diagnosis, intervention, and treatment are critical for controlling the genetic hearing loss. Although many studies indicated that, similar to Western countries, mutations in *GJB2, SLC26A4,* and *mtDNA 12S rRNA *(MIM #561000) are the major cause of the genetic deafness in China (Pan et al., 2017), the molecular etiology of non‐syndromic deafness in the Chinese population has not been systematically investigated. In this study, in order to investigate the molecular etiology of non‐syndromic deafness patients from Shanxi Province, we have performed mutation analysis of three common deafness‐related genes (*GJB2, SLC26A4*, and *mtDNA 12S rRNA*) in 1,201 patients with non‐syndromic deafness in Shanxi Province, which has a high incidence of HL. Our data suggested that 21.23%, 9.33%, and 1.25% of the patients carry mutations in *GJB2*, *SLC26A4*, and *MT‐RNR1* in Shanxi Province. These data will enrich the database of deafness mutations and provide the standard for clinical diagnosis, treatment, and genetic counseling in Shanxi. Also, it will contribute to development of a diagnosis kit for patients with non‐syndromic hearing loss in Shanxi Province.

## 2 MATERIALS AND METHODS

### 2.1 Ethical compliance

This study was approved by the ethics committee of the Second Hospital of Shanxi Medical University.

### 2.2 Subject information

In total, 1,201 non‐syndromic deafness subjects from Shanxi Province, who were enrolled from the School for deaf‐mute and special school, were recruited. Family history was obtained by consulting subjects or their parents. Individuals of the control group without family history were enrolled from the Blood Transfusion Department of the Second Hospital of Shanxi Medical University. In the patient group, there were 698 males and 503 females, with an average age of 19.25 years, ranging from 0.5 to 38 years. Both patients and control individuals were Han Chinese and have signed informed consent by themselves or their parents according to the protocol approved by the Ethics Committee of the Second Hospital of Shanxi Medical University.

### 2.3 Deafness questionnaire survey

All patients or their guardians completed the survey by answering questions and filling in the "deafness questionnaire survey form." The details were as follows: (a) basic information: name, gender, date of birth, nationality, marital status, contact information, etc.; (b) morbidity status: age of onset, presence of other associated symptoms (vertigo, tinnitus, etc.), related medical history (infectious diseases, etc.), history of use of aminoglycoside antibiotics, long‐term noise exposure, otitis media or traumatic history of the ear, etc.; (c) the development condition of language, whether wearing hearing aid or cochlea, etc.; and (d) whether merging other systemic diseases (eyes, bones, intelligence, etc.), family history, and eclampsia pregnancy history: the infecting status of mothers during pregnancy (cytomegalovirus, ulcer, syphilis, rubella, etc.) or the presence or absence of dystocia.

### 2.4 Clinical evaluation

Ear examination and audiological evaluations including pure‐tone audiometry, immittance testing, auditory brainstem response, and auditory steady‐state response were performed for all hearing impairment patients. Physical and neurological examination was carried out with special attention to renal and ophthalmologic differences to exclude those with syndromic deafness. Only non‐syndromic deafness subjects were kept in the study.

### 2.5 Mutation analysis

The mutations of three deafness genes (*GJB2*, *SLC26A4, *and *mtDNA rRNA*) were investigated in all the subjects. Genomic DNA was extracted from the peripheral blood using QIAGEN Blood DNA Kits (Qiagen Biotech Co., Ltd, Shanghai, China) following the manufacturer's instructions. Gene‐specific primers for amplifying exons of *GJB2 *(NM_004004.5) and parts of *SLC26A4* (NM_000441.1) were designed by Primer 5.0 software, and the sequences are listed in Table 1; other parts of *SLC26A4* (NM_000441.1) were sequenced using the conditions reported by Sagong, Baek, Baek, Lee, and Kim (2017). The exons were amplified by PCR with the following conditions: initial denaturing phase at 94°C for 3 min, followed by 35 repetitions at 95°C for 40 s, Tm for 40 s, and 72°C for 40 s, with a final extension for 7 min at 72°C; and the PCR products were stored at 4°C. The PCR products were verified by agarose gel electrophoresis (2% agarose) and then purified using the QIAquick PCR Purification Kit (Qiagen Biotech Co., Ltd). The Sanger sequencing was performed by Shanghai Sangon Biotech Co., Ltd, and the mutations were analyzed by Mutation Surveyor software.

**Table 1:** Deafness gene primer sequences

Gene	Primer sequence	Length (bp)	*T*_m_ (°C)
*GJB2* ^a^	F: CCAGGCTGCAAGAACGTGTG R: TGAGCACGGGTTGCCTCATC	574	62
*SLC26A4 7+8* ^b^	F: CGTGTAGCAGCAGGAAGTAT R: TTAAATAAAAAAGACTGACT	443	55
*SLC26A4 9* ^a^	F: TGGGGAAAAAGGATGGTGGT R: CCAACCCCTTCTTTAGCTGA	344	56
*SLC26A4 16* ^a^	F: GCAGGATAGCTCAAGGAATT R: TCATCAGGGAAAGGAAATAA	262	55
*SLC26A4 17* ^b^	F: TCTCCTTGATGTCTTGCTTA R: CCCATGTATTTGCCCTGTTG	598	58
*SLC26A4 19* ^b^	F: CTGGGCAATAGAATGAGACT R: ATCTGTAGAAAGGTTGAATA	260	55
*mtDNA 12S rRNAACCCCC*	F: CCGCCATCTTCAGCAAACCCTG R: TAGTAAGGTGGAGTGGGTTTGG	444	59

^a^Gene‐specific primers for amplifying exons of *GJB2 *(NM_004004.5) and ^b^parts of *SLC26A4* (NM_000441.1) were designed by Primer 5.0 software.

### 2.6 CT scan

Patients carrying *SLC26A4* mutations were examined by temporal bone computed tomography (CT) for diagnosis of enlarged vestibular aqueduct (EVA) or inner ear malformation. The diagnosis standard is diameter >1.5 mm at the midpoint between the common crus and external aperture (Sagong et al., 2017; Zhu et al., 2017). The CT scan was performed at the CT Department of The Second Affiliated Hospital of Shanxi Medical University. The results were analyzed by the experts of the Otolaryngology Department.

## 3 RESULTS

After investigation and inquiry, 936 patients (77.94%) were found with hearing loss under the age of 3 years (they were prespeech deafness). Forty‐seven cases of deafness (3.91%) were found after 3 years old (they were postspeech deafness). Thirty‐four patients were found with hearing loss after high fever, accounting for 2.83%. Twenty‐three patients provided a history of the usage of aminoglycoside drugs. Sixty‐three patients were infected with CMV, and eight cases had HL, accounting for 12.7% (8/63). Seventy‐one patients had the history of injury, resulting in HL. There were 27 cases of deafness with unclear medical history. In the 1,201 patients with non‐syndromic deafness, 1,146 were full‐frequency hearing loss, 38 were high‐frequency hearing loss, and 17 were low‐frequency hearing loss. There were 786 cases of binaural hearing impairment and 415 cases of monaural hearing impairment. Immittance testing revealed that all patients evoked otoacoustic emissions.

**Figure 1:**
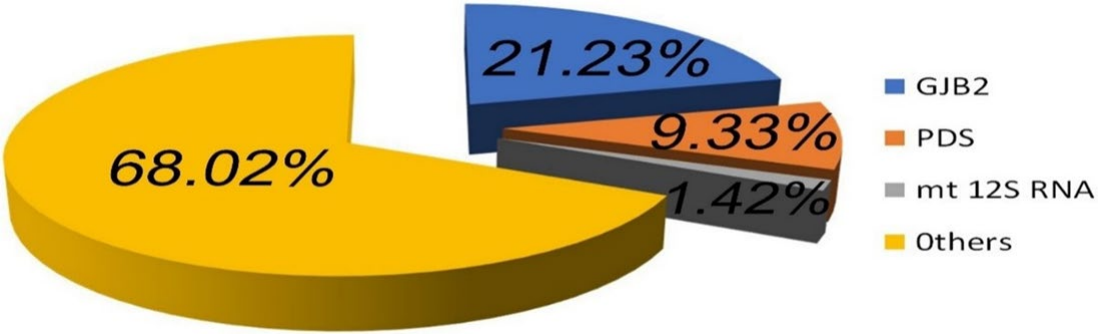
Distribution of mutations of the three common deafness genes in 1,201 patients in Shanxi Province. In the patient group, *GJB2* mutations accounted for 21.23% (255/1,201) and *SLC26A4* mutations accounted for 9.33% (112/1,201). In addition, 15 patients (1.25%) were found to carry *12S rRNA* m.1555A>G mutation, while only two cases carried *12S rRNA* m.1494C>T. Others have not found mutations in the three common genes

Among the 1,201 patients recruited in this study, *GJB2* mutations were found in 21.23% (255/1,201) of the patients; *SLC26A4* mutations were found in 9.33% (112/1,201) of the patients; and *12S rRNA* mutations were found in 1.42% (17/1,201) of the patients. In 17 *mtDNA 12S rRNA* mutation subjects, 15 carried the m.1555A>G and two carried the m.1494C>T (Figure 1).

### 3.1 *GJB2* mutations

**Figure 2:**
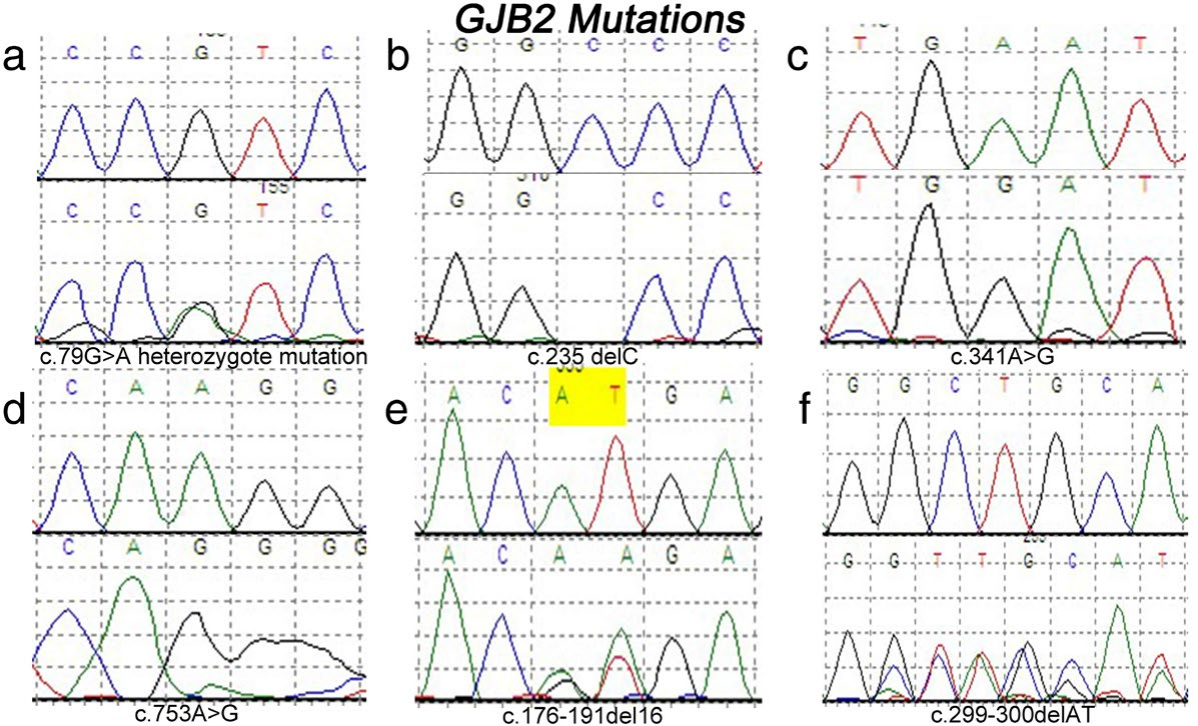
Common mutations of *GJB2*. Top: sequence in the control group; and bottom: sequence in the patient group (a) c.79G>A heterozygote mutation.(b) c.235 delC. (c) c.341A>G. (d) c.7753A>G. (e) c.176‐191del16. (f) c.299‐300delAT

In total, 257 mutations were detected, with 255 found in the patient group and two in the control group. As shown in Table 2, c.235delC, c.176‐191del16, c.299‐300delAT, c.109G>A, and c.35delG were the most frequent mutations found in this study. c.235delC was the most frequent mutation in non‐syndromic hearing loss (NSHL) in Shanxi, accounting for 10.99% (132/1,201). The second frequent mutation is c.299‐300delAT, accounting for 2.67% (32/1,201). Three other mutations (c.79G>A heterozygous mutation, c.341A>G, and c.753A>G) were analyzed, as shown in Figure 2. Notably, ten new mutations in *GJB2* were detected in the patient group (Table 3). Their function remains to be further studied.

**Table 2:** High‐frequency mutation of three common deafness genes

Gene	Mutations	*N*		Frequency (%)
		Heterozygote	Homozygote	
*GJB2*	c.176‐191del16	12	0	1.00
	c.235delC	76	56	10.99
	c.109G>A	16	9	2.08
	c.299‐300delAT	29	3	2.67
	c.35delG	8	0	0.67
*PDS*	c.919‐2A>G	41	16	4.75
	c.1229C>T	1	0	0.08
	c.2168A>G	3	8	1.67
*mt12rRNA*	m.1555A>G	0	15	1.25
	m.1494C>T	0	2	0.17

**Table 3:** New mutations of three common deafness genes

Gene	Mutations	*n*		Frequency (%)
		Heterozygote	Homozygote	
*GJB2*	c.8906‐8907insAAGG	1	0	0.08
	c.54 C>A	4	1	0.42
	c.88A>G	1	0	0.08
	c.127G>C	1	0	0.08
	c.186C>T	1	0	0.08
	c.187G>T	1	0	0.08
	c.199C>A	0	1	0.08
	c.277A>G	1	0	0.08
	c.319A>G	1	0	0.08
	c.558G>A	1	0	0.08
*PDS*	IVS18‐78G>A	2	0	0.17
	c.2095G>A	6	8	1.17
	c.2107C>G	1	0	0.08
	c.2134A>T	0	2	0.17
	c.2135A>T	2	1	0.25
	c.2136C>T	1	0	0.08
	c.2137A>G	2	0	0.17
	c.2149A>C	0	1	0.08
	c.IVS19+18C>A	2	0	0.17

In patients with *GJB2 *mutation, 16.90% (203/1,201) carried a single mutant allele and 4.33% (52/1,201) carried two mutant alleles. Four patients showed compound heterozygous mutations carrying c.35delG;c.235delC, c.176‐191del16; c.299‐300delAT, c.35delG;c.176‐191del16, and c.235delC;c.299‐300delAT, respectively.

Among the 300 healthy individuals, only two subjects were found to carry heterozygous pathogenic mutation c.235delC with a frequency of 0.67% (2/300); two other variants, c.79G>A and c.341A>G, accounted for 28.67% (86/300) and 14.00% (42/300), respectively. c.79G>A and c.341A>G were polymorphisms that could lead to nonsynonymous changes: p.V27I and p.E114G, respectively (Yuan et al., 2012).

### 3.2 *SLC26A4* mutations

**Figure 3:**
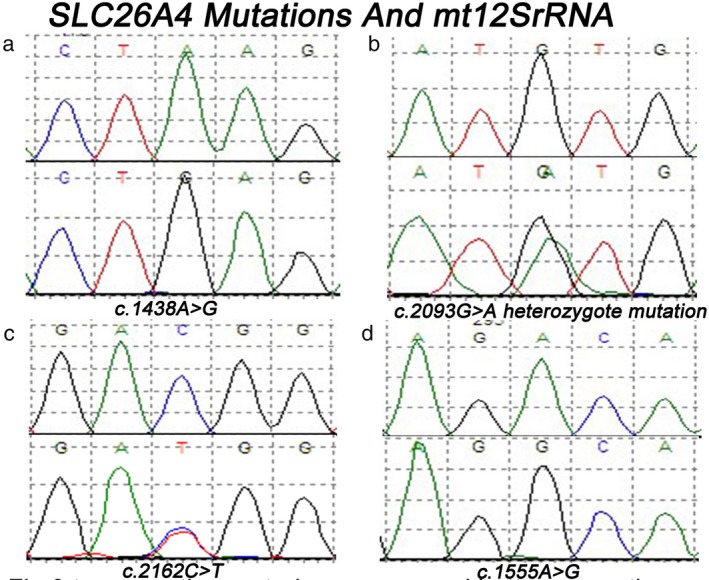
Common mutations of *SLC26A4 and mt12SrRNA*. Top: sequence in the control group; and bottom: sequence in the patient group (a) c.1438A>G. (b) c.2093G>A heterozygote mutation. (c) c.2162C>T. (d) c.1555A>G

Mutations in *SLC26A4* were detected in the 1,201 patients (Figure 3). c.919‐2A>G was found in 57 patients and c.2168A>G was identified in 20 patients.This study revealed that 2.66% (32/1,201) of the patients had two mutant alleles. But Five patients with severe hearing loss with EVA were found to be compound heterozygotes for c.919‐2A>G and c.2168A>G. No patients carried a single mutant allele of *SLC26A4*. Three individuals in the control group were detected to carry c.919‐2A>G. This study revealed that 2.66% (32/1,201) of the patients had two mutant alleles (homozygote or compound heterozygote).

### 3.3 *mtDNA 12S rRNA* mutation

As the *mt12S rRNA* gene mutation was reported as one of major causes of non‐syndromic deafness, the mutation of this gene was investigated (Figure 3). Fifteen patients with non‐syndromic deafness were found to carry c.1555A>G, while two cases carried *mtDNA 12S rRNA* c.1494C>T. No mutation of *mt12S rRNA *gene was found in the control group.

## 4 DISCUSSION

Hearing loss is one of the most common neurosensory disorders in humans. It is a worldwide prevalent disorder and severely affects human life quality. Approximately one to three in 1,000 children suffer HL at birth or during early childhood. Non‐syndromic deafness is the most common deafness, accounting for 70% of hearing impairment. In patients with non‐syndromic hearing loss, 50% of cases were related to genetic mutation (Yan et al., 2017). An important percentage of the hearing impairments are expected not to be of genetic origin and linked, for example, to CMV infection, other environmental factors, or a combination of genetic and environmental factors. Thus, it is very important to explore the gene mutations associated with deafness for early diagnosis and treatment. However, there is no comprehensive study of gene mutations associated with non‐syndromic deafness in the Chinese population. In our study, we have investigated the frequency of three common deafness‐related genes, *GJB2, SLC26A4, *and *mtDNA 12S rRNA*, in the patients with non‐syndromic hearing loss from Shanxi Province, an area with a high birth defect rate.

In all 1,201 patients and 300 control individuals, mutations in *GJB2* were detected; c.235delC was the most common mutation with a frequency of 10.99%, slightly lower than other reports in the Chinese population (Li et al., 2017; Ma et al., 2016; Pan et al., 2017). Together with our results, the c.235delC mutation in *GJB2* shows the highest frequency in the Chinese population, followed by c.299‐300delAT with a frequency of 2.67% (32/1,201). It was reported that *GJB2* mutations are also associated with a large proportion of non‐syndromic deafness patients in Austria and Africa (Parzefall et al., 2017; Rudman et al., 2017). Twenty‐five patients were found to carry heterozygous mutation of *GJB2* c.109G>A, accounting for 2.08% (25/1,201). However, this mutation was not found in the 300 healthy individuals. A previous study has shown that c.109G>A was a polymorphism (Yuan et al., 2012), but another study claimed that it is pathogenic. In the study, compound heterozygous mutations in *GJB2* were found in four patients. Recent evidence suggests that the *GJB2* genotype plays an important role in non‐syndromic hearing loss (Laleh et al., 2017). A study by Huang et al. also indicated that the hearing loss of patients with *GJB2* mutation was more severe than that of those patients carrying *SLC26A4* or *12S rRNA* mutations (Huang et al., 2013). This result was also demonstrated in our study.

A total of 112 (9.33%) cases in the patient group and three individuals in the control group were found to carry mutations of *SLC26A4*. Similar to other studies, our study revealed that c.919‐2A>G and c.2168A>G were the most frequent mutations of *SLC26A4* in Shanxi Province (Adhikary et al., 2015; Fang et al., 2015; Ma et al., 2016). *SLC26A4* mutations are strongly associated with inner ear malformation and EVA. It can be definitely diagnosed by temporal bone CT. Individuals with this mutation should avoid strenuous exercise, trauma, and collision. In our study, imageological examination was performed in 112 patients with *SLC26A4* mutations and all were verified with EVA.

It has been estimated that approximately 20% of inherited postlingual deafness may be connected with mutations in the mitochondrial genome, but large ethnic variations might be present (Fan, Zhu, Tang, & Xue, 2017; Mutai et al., 2017). Fifteen patients were found to carry the c.1555A>G homoplasmic mutation in this study, accounting for 1.25% (15/1,201). The frequency of *mtDNA 12S rRNA* mutations found in the present study was lower than either that reported by Guo et al. (2008) in northern Chinese subjects or that reported in the northwest of China (Ma et al., 2016; Zhu et al., 2015). This result may be related to the fact that a majority of our patients had congenital and prelingual deafness. Patients with *mtDNA 12S rRNA *mutations should avoid using aminoglycoside, since it will induce HL (Dai et al., 2008).

Of the 1,201 patients in the patient group, 63 were infected with CMV, of which eight had HL, accounting for 12.7% (8/63). This suggests that CMV infection is an important factor in the occurrence of deafness, but its mechanism has not been fully elucidated at present. Most scholars believe that it mainly causes deafness by the development of inhibiting sensory nerve.

## 5 CONCLUSION

In our research, it was found that c.235delC in *GJB2* and c.919‐2A>G in *SLC26A4 *were the most prevalent pathogenic variants in Shanxi Province. This result is consistent with previous reports. The cause of deafness is very complex. Deafness is influenced by genetic and environmental factors. It is an arduous task to identify the pathogenic mechanism. However, more and more genes related to deafness were found. Epidemiological studies revealed that the mutation frequency of the three common deafness genes varies in different regions. The results of our research will lay a foundation for the prevention of deafness in Shanxi Province. They will enrich the database of deafness mutations and make a contribution to clinical diagnosis, treatment, and genetic counseling in non‐syndromic hearing loss in future.

## CONFLICT OF INTEREST

None declared.
